# Sandwich parastomal hernia repair, a prospective observational study

**DOI:** 10.1007/s10029-025-03335-6

**Published:** 2025-06-21

**Authors:** T. B. Johnsen, T. Stornes, B. Ystgaard, T. E. Bernstein

**Affiliations:** 1 Department of Surgery, St. Olavs Hospital, Trondheim University Hospital, Pb 3250 Torgarden, Trondheim, 7006 Norway; 2 Department of Surgery, St. Olavs Hospital, Trondheim University Hospital, Trondheim, Norway; 3 Institute of Clinical and Molecular Medicine, Norwegian University of Science and Technology, Trondheim, Norway

**Keywords:** Ostomy, Hernia, Parastomal hernia, Treatment, Recurrence

## Abstract

**Purpose:**

The aim of the study is to evaluate the safety, feasibility and short and long term outcomes following the Sandwich repair for parastomal hernia.

**Methods:**

A prospective single center study including all patients operated with Sandwich repair at St. Olav hospital, Trondheim University hospital from 2018 to 2023.

**Results:**

Fifty four patients were treated with the Sandwich repair. All procedures were performed laparoscopically, with two conversions to open surgery due to adhesions. The median age was 67 years. 44% of the patients were females. Two thirds of the patients had a colostomy. Pain or discomfort was the most frequent indication for parastomal hernia repair, followed by leakage. Median operation time was 149 min. Eight patients were operated in an emergency setting. Six patients had a recurrence after previous surgery for parastomal hernia. Three patients had ClavienDindo complication rate 3b following the Sandwich repair and had their implants removed a few days after index surgery. The median in-hospital time was 5 (2–35) days and the median followup was 33 months (1–61). No recurrences were identified.

**Conclusion:**

The laparoscopic Sandwich repair is a safe methode for most of the parastomal hernia patients. Even with a reasonable long median follow-up, we did not observe any recurrences.

## Introduction

Patients with an ostomy have a risk for development of parastomal hernia. The incidence is increasing with time, depending on type of stoma and patient characteristics. It is estimated that for colostomies the incidence is more than 30% after 12 months, and 50% with a follow- up of more than 2 years [1, 2]. A significant proportion of these patients will have symptoms requiring surgery, both in planned and emergency settings. Repair for parastomal hernia lacks international guidelines and due to insufficient data there is no scientific basis for recommending an optimal technique [3].

The recurrency rates for various repair has been high throughout. Ostomy re-location and direct suture has recurrence rates ranging between 0 and 72% and 46–100%, respectively [4–6]. Following the introduction of laparoscopic mesh repairs, e.g. Modified Sugarbaker and Keyhole technique, the recurrence rates have improved somewhat. Still, for these two methods, the recurrence rates remain high, 9% and 24.1%, respectively [7].

Dieter Berger was the first to combine the Keyhole and the Modified Sugarbaker techniques (Sandwich technique) and published a single center study in 2007 with only 2.1% recurrence rate [8]. Our technique is based directly on Dieter Bergers, and since 2012, the Sandwich technique has been the primary method for parastomal hernia repair. The method was revised in 2018 following the withdrawal of the prefabricated mesh (Covidien Parietex™ Composite Parastomal Mesh(PCOPM15)) used for this purpose.

The aim of this study was to evaluate the safety of the Sandwich method and presenting short- and long-term results including recurrence after this surgery.

## Materials and methods

This study includes all patients undergoing planned or emergent parastomal hernia repair with the Sandwich technique from 1 January 2018 to 1 January 2024.

All data were prospectively registered, and the study was approved in the regional ethics committee (REK no. 683354).

Data include baseline characteristics and comorbidity, previous surgery, type of ostomy, indications for primary operations, indications for hernia repairs, types of operation, operative data and postoperatice complications. Follow-up data were recorded prospectively. Complications were graded according to the Clavien-Dindo scoring system [9] and the hernias was classified according to the European Hernia Society (EHS) classification [10].

A re-operation was defined as any surgical intervention within 100 days of index surgery. All the procedures were performed by three surgeons. The procedure has become strictly standardized, thus following the same stepwise sequence of elements for every patient: Patients were in supine position. Arms were tucked in at the sides. All patients were given prophylactic antibiotics (Cefazolin 2 gr x 3). Pneumoperitoneum was established with Visiport alone or combined with Verres needle. The three ports were placed in the opposite flank of the stoma occasionally an extra 5 mm port was inserted. If adhesiolysis was needed it was done with scissiors or Ligasure. The stoma was then mobilized so the bowl could be lateralized. The defect in the abdominal wall was closed with Novafil 0 sutures using endoclose. The abdominal pressure was reduced to 5 mm Hg before closing. The Key hole mesh is placed and secured with a lateral transfix suture (Prolen 2 − 0) and medial transfix suture (Prolen 2 − 0) brought through both medial flaps. We use Endoclose to place the transfix sutures. All patients were operated with 35 mm opening in the keyhole mesh. The bowl is lateralized with a 20 cm Symbotex composit mesh. A 15 × 6 cm Symbotex composit mesh is sutured to the Sugerbaker mesh with the film against the stoma. This to prevent in-growth. Two transfix sutures are placed lateral. One on each side of the stoma. They are placed 4–5 cm from each other to prevent obstruction. Medial the Sugerbaker mesh is fixated with a transfix suture (Prolen 2 − 0). The sugerbaker mesh is then fixated with dual crown absorbable tackers.

For the keyhole mesh we used Parietex composite mesh which is a monofilament mesh coated with a protective collagen-based film to prevent in-growth into the bowel. The mesh for lateralization of the intestine was constructed with a Symbotex composite mesh 20 cm in diameter.

Most patients with urostomi were excluded as the lateralization was considered difficult when applying a circular mesh of 20 cm in diameter. From 2022 the parastomal hernias in the urostomy patient were operated with a retromuscular plasty. This methode will be discussed in a separate paper. In the time frame of the study 74 patients were operated for parastomal hernias. 54 had a Sandwich plasty.

As the Sandwich plasty is considered an IPOM (IntraPeritoneal Onlay Mesh) procedure, we did not apply this methode for any patient with Mb. Crohn in order to avoid the exposure between mesh and intestine. Furthermore, patients expected to have frozen/hostile abdomen due to previous multiple operations or infections, were operated with a retromuscular plasty as menshioned previously in order to avoid entering laparoscopically.

All patients were followed up in the outpatient clinic after 3 and 12 months and then annually for a total of 5 years. Data was registrered in a secure database, and analyzed with the use of SPSS statistics version 29 (IBM data).

## Results

Fifty-four patients were included in the study, Table 1. All procedures were performed and completed laparoscopically, except in two patients in which the operations were converted to open surgery due to adhesions. The study included 24 women and 30 men. Median age was 67 years (Range 23–85), and median BMI was 27.8 (Range 19.2–37.9). Twenty eight of the 54 patients were classified as ASA 3. There were no ASA 4. Thirty five patients had a colostomy, 18 ileostomy and 1 urostomy, All patients had to stop smoking 3 months ahead of surgery. Two of the patients later reported that they did not.

**Table 1:**
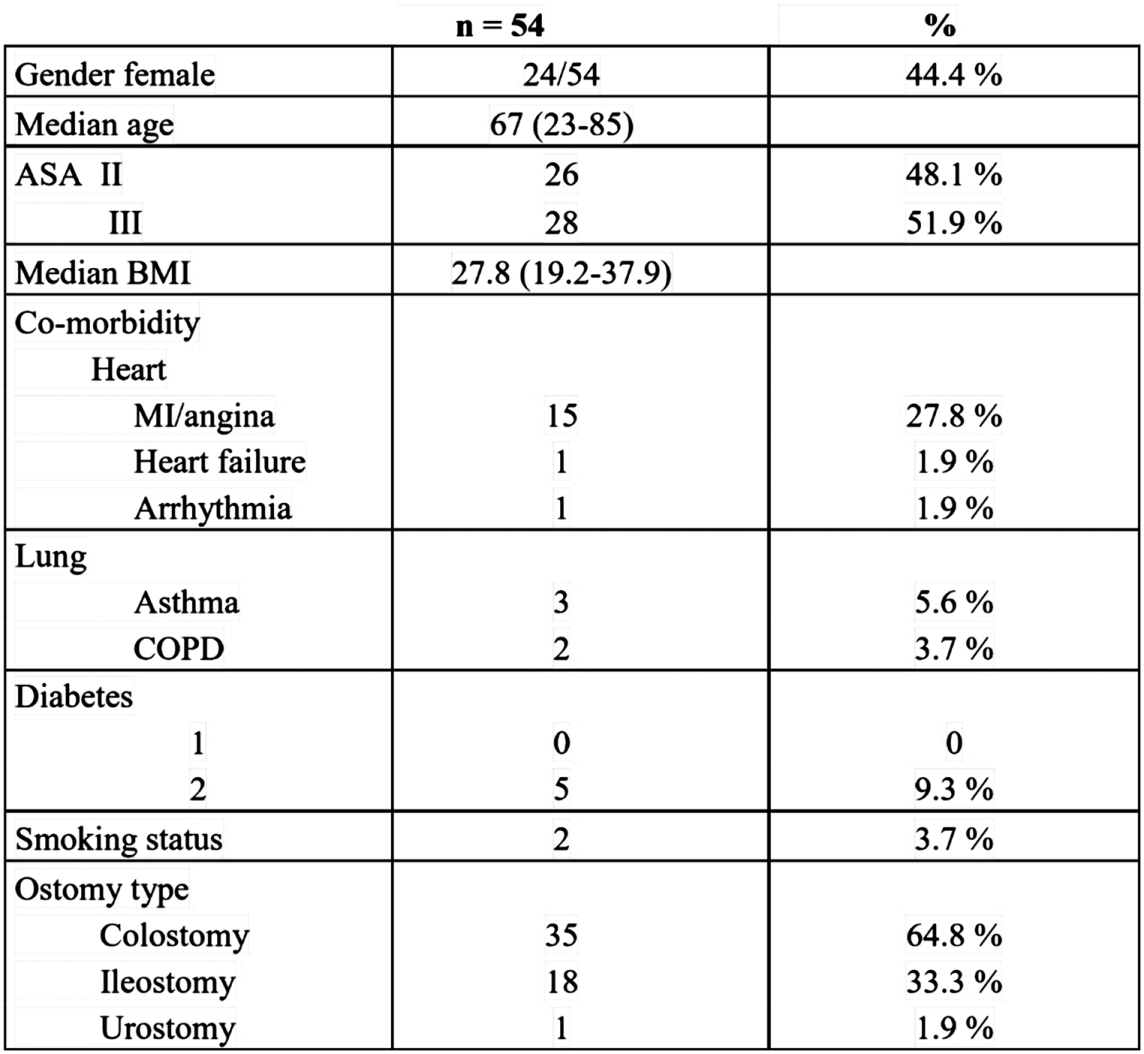
Characteristics of 54 patients operated with sandwich plasty

The indications for the primary ostomy formation are given in Table 2.

**Table 2:** Indication for primary ostomy operation (*n* = 54)

	*n*	%
Ulcerative colitis	17	31.5
CR-Cancer	18	33.3
Colorectal functional disorders	18	33.3
Urological functional disorder	1	1.9

No patient had morbus Crohn in the IBD group. The indication for ostomy among patients within the functional disorder group, included 2 patients with rectal prolaps, 6 patients with incontinence, 2 patients with solitary rectal ulcer syndrome (SRUS), 4 patients with diverticulitis, one with obstructive defecation syndrome (ODS), and 3 with constipation. One patient with Bricker deviation, after urine leakage following prostatectomy developed parastomal hernia and due to a long segment it was possible to lateralize it sufficiently for a Sandwich repair. Twenty six of the patients had previously at least one laparotomy, and 6 of them had 2 laparotomies or more.

The primary indications for parastomal hernia repair are given in Table 3.

**Table 3:** Indications for parastomal hernia repair (*n* = 54)

	*n*	%
Pain	36	66.7
Leakage	10	18.5
Obstruction	4	7.4
Strangulation	1	1.9
Protrusion of ostomy	3	5.6

66% of the patients had more than one indication for a parastomal hernia repair. The median duration of the procedure was 149 (76–230) minutes.

Eight patients had surgery in an emergent setting. Six of the 54 patients had a reccurrent parastomal hernia. One of these also had a concomitant incisional hernia. Table 4 shows the grading of the hernias according to the European Hernia Society (EHS)classification.

**Table 4:** Classification of the 54 parastomal hernias according to EHS classification (type I-IV)

Concomitant incisional hernia	Small ≤ 5 cm	Large > 5 cm
No	42 (Type I)	1 (Type III)
Yes	4 (Type II)	7 (Type IV)

Median length of hospital stay was 5.5 (2–35) days. Twelve patients had postoperative complications, with Clavien-Dindo classification given in Table 5. Three patients had to be reoperated and have the meshes removed shortly after the primary procedure due to complications. The 100 days re-operation rate was 5.6%. One of these patients had a perforated diverticula in the lateralized part of the colon, the second one had the lateralized part of the colon perforated following the insertion of a deflatation tube for alleviating postoperative retention, and the last developed ischemia in the lateralized intestine the day after the Sandwich procedure. Following the removal of the mesh, the first underwent a long-course VAC treatment and subsequently developed an incisional and a parastomal hernia. The patient was in the end successfully operated with a new mesh repair. The other two patients were definetly treated by changing the ostomy side. No surgical site infection occured in the other patients within 30 days of operation.

**Table 5:** Types of complications and classification acording to Clavien-Dindo

	Clavien - Dindo				
Types of complications	n	1	2	3a	3b
Retention	2	1			1
Constipation	2	2			
Seroma	3	2		1	
Pneumonia	1		1		
Urinary retention	1			1	
Perforation	2				2
Ischemia	1				1

On discharge, 82% of the patients still was on opioids on demand. Only one patient used opiods after 3 months.

One patient originally operated for rectal cancer died due to metastatic disease 37 months after the Sandwich repair. Three patients died after 12, 15 and 38 months for reasons unrelated to the Sandwich surgery.

One patient was readmitted 5 years after Sandwich repair due to intestinal obstruction treated with adhesiolysis in open surgery.

No reccurences was observed, with a median follow-up of 33 months. Four patients were followed for more than 60 months.

## Discussion

The most important finding in this study is the complete absence of parastomal hernia recurrence following Sandwich repair. As the present article includes 54 patients it is so far the largest study on this technique since the original work of Dieter Berger [8].

The surgical techniques for parastomal hernia repair have traditionally been associated with high recurrence rates [4–7]. Later, in spite of a significant improvement after the introduction of different IPOM mesh techniques, such as the keyhole and the modified Sugarbaker, the recurrence rates between 9 − 24.1% have still been too high [7]. Today, the laparoscopically modified Sugarbaker repair is the preferred and most widespread method due to lower rate of recurrences. The original paper from Berger in 2007 reported a recurrence rate of 2.1%. The present study with no recurrences has confirmed that the Sandwich repair is our best methode and our results underline that the procedure can be recommended.

With the keyhole mesh alone, the recurrence appears as the keyhole opening increases in size with time [11, 12]. With the modified Sugarbaker mesh, recurrences appears laterally as the abdominal wall ventral to the lateralized intestine lacks the support of the mesh [8]. Using the meshes in combination addresses both these problems. The modified Sugarbaker mesh will prevent any protrusion of intraabdominal structures through the keyhole opening. The keyhole mesh will stabilize the abdominal wall ventral to the lateralized intestine, thus preventing the lateral recurrence. It is of outmost importance that the two meshes are aligned properly in the relation to each other. As the circular key-hole mesh is 15 cm, the 20 cm Sugarbaker mesh has to cover the key hole mesh completely with a 2.5 cm brim peripherally and circumferentially. The brim of 2.5 cm will incorporate into the abdominal wall, as will the more centrally part of the Sugarbaker mesh as it is secured via the key hole mesh into the abdominal wall by tackers and transfixation sutures.

A strict focus on standardization of the surgery adhering to the sequential steps of the procedure is mandatory for high quality. Furthermore, the procedures in the present study have been performed by only three surgeons.

The median follow-up in the present study was 33 months (follow up range 3–60 months). For comparable studies using laparoscopic IPOM procedures the mean follow-up varied between 14 and 36 months [13, 14], and median follow-up between 19 and 36 months [15]. However, most of the studies had a follow up under 25 months [16–18]. A recent study including 38 patients operated with Sandwich repair, had a median follow-up of 39 months and reported a recurrence rate of 7.9% [19]. This recurrence rate was somewhat higher than the recurrence rate of 3.5% presented in the meta-analysis from 2023 [7] bases on 5 studies on Sandwich repair [8, 20, 21, 22, 23].

A recent study on 26 robotic Pauli procedures from 2022 with a median follow-up of 14 months (0–30) reported a recurrence rate of 3.8% (1/26) [24]. Similarily, another publication from 2021 also with a 14 months follow-up including 15 patients with robotic modified Sugarbaker had a recurrence rate of 6–7% (1/15) [25].

The present study indicates that the procedure is suitable in most situations of BMI, ASA, previous operations, different types of hernias including recurrent hernias and even in emergency setting.

The BMI of 28 in the present study is comparable with the BMI of 28 in the publication on.

Sandwich repair from 2007 and the BMI of 27 in the study on robotic Pauli procedures from 2022. The median operating time was 115 min in the study from 2007, compared to 149 min in the present study.

In the study from 2007 the median postoperative hospital stay was 10 days (5–67) in contrast to 5.5 days (2–35) in the present study. The meta-analysis from 2023 reported 6 and 9.7 days, respectively [7]. The patients operated with the robotic Pauli procedure had a median hospital stay of 3 days (1–13).

In the present study, 22% of the patients experienced complications to some degree. This included 7% with Clavien-Dindo 3b, needing a new procedure in general anesthetics. One of these had an endoscopy through the stoma and the rest had the mesh removed as outlined in the Result section. The rest of the patients with complications were classified as 3a or less. Except for the three patients with removal of meshes shortly after the operation, there were no SSI. Among the robotic Pauli patients there were 31% postoperative complications.

The present study only included one urostomy. Usually, the Bricker urostomy is 15 cm long. With a 20 cm circular mesh for lateralization, a normal Bricker will not fit into this repair. This was also seen in the r-Pauli study, where only one patient with urostomy was included [24].

The Sandwich method is our primary choice for parastomal hernia repair. However, in certain situations where lateralization is not possible or entrance into the abdomen, are expected to be difficult or associated with a high risk, we will choose another method involving an open sublay mesh technique, which will be presented in a later publication in detail.

In conclusion, the laparoscopic Sandwich repair is considered a safe procedure, with a low recurrence rate and an acceptable complication rate. The combined properties of the two separate implants working together makes the Sandwich procedure superior to one-mesh procedures.

## References

[1] Sohn YJ et al (2012) Incidence and risk factors of parastomal hernia. J Korean Soc Coloproctol 28(5):241–246 23185703 10.3393/jksc.2012.28.5.241 PMC3499424

[2] Israelsson LA (2008) Parastomal hernias. Surg Clin North Am 88(1):113–125 18267165 10.1016/j.suc.2007.10.003

[3] Antoniou SA et al (2018) European hernia society guidelines on prevention and treatment of parastomal hernias. Hernia 22(1):183–198 29134456 10.1007/s10029-017-1697-5

[4] Etherington RJ et al (1990) Demonstration of para-ileostomy herniation using computed tomography. Clin Radiol 41(5):333–336 2354601 10.1016/s0009-9260(05)81696-4

[5] Rubin MS, Schoetz DJ Jr, Matthews JB (1994) Parastomal hernia. Is stoma relocation superior to fascial repair? Arch Surg 129(4):413–418 8154967 10.1001/archsurg.1994.01420280091011

[6] Riansuwan W et al (2010) Surgery of recurrent parastomal hernia: direct repair or relocation? Colorectal Dis 12(7):681–686 19486097 10.1111/j.1463-1318.2009.01868.x

[7] Kritharides N et al (2023) Laparoscopic parastomal hernia repair: keyhole, Sugarbaker, sandwich, or hybrid technique with 3D mesh? An updated systematic review and metaanalysis. Langenbecks Arch Surg 408(1):448 38017096 10.1007/s00423-023-03177-9 PMC10684625

[8] Berger D, Bientzle M (2007) Laparoscopic repair of parastomal hernias: a single Surgeon’s experience in 66 patients. Dis Colon Rectum 50(10):1668–1673 17680310 10.1007/s10350-007-9028-z

[9] Dindo D, Demartines N, Clavien PA (2004) Classification of surgical complications: a new proposal with evaluation in a cohort of 6336 patients and results of a survey. Ann Surg 240(2):205–213 15273542 10.1097/01.sla.0000133083.54934.ae PMC1360123

[10] Śmietański M et al (2014) European hernia society classification of parastomal hernias. Hernia 18(1):1–6 24081460 10.1007/s10029-013-1162-z PMC3902080

[11] Hansson BM, Bleichrodt RP, de Hingh IE (2009) Laparoscopic parastomal hernia repair using a keyhole technique results in a high recurrence rate. Surg Endosc 23(7):1456–1459 19118435 10.1007/s00464-008-0253-x

[12] Muysoms F (2007) Laparoscopic repair of parastomal hernias with a modified sugarbaker technique. Acta Chir Belg 107(4):476–480 17966555 10.1080/00015458.2007.11680104

[13] Pastor DM et al (2009) Parastomal hernia repair: a single center experience. JSLS 13(2):170–175 19660211 PMC3015921

[14] Craft RO et al (2008) Laparoscopic parastomal hernia repair. Hernia 12(2):137–140 17999128 10.1007/s10029-007-0299-z

[15] Mancini GJ et al (2007) Laparoscopic parastomal hernia repair using a nonslit mesh technique. Surg Endosc 21(9):1487–1491 17593454 10.1007/s00464-007-9419-1

[16] LeBlanc KA et al (2005) Laparoscopic parastomal hernia repair. Hernia 9(2):140–144 15602627 10.1007/s10029-004-0295-5

[17] McLemore EC et al (2007) Parastomal hernia: short-term outcome after laparoscopic and conventional repairs. Surg Innov 14(3):199–204 17928619 10.1177/1553350607307275

[18] Safadi B (2004) Laparoscopic repair of parastomal hernias: early results. Surg Endosc 18(4):676–680 15026932 10.1007/s00464-003-8518-x

[19] Barranquero AG et al (2023) Analysis of recurrence and risk factors in laparoscopic sandwich technique for parastomal hernia repair. Surg Endosc 37(12):9125–9131 37814164 10.1007/s00464-023-10475-2

[20] Mäkäräinen-Uhlbäck E et al (2021) Parastomal hernia: A retrospective nationwide cohort study comparing different techniques with Long-Term Follow-Up. World J Surg 45(6):1742–1749 33560501 10.1007/s00268-021-05990-z PMC8093171

[21] Köhler G et al (2015) Changes in the surgical management of parastomal hernias over 15 years: results of 135 cases. World J Surg 39(11):2795–2804 26264458 10.1007/s00268-015-3187-1

[22] Hashida H et al (2021) Analysis of the outcome of laparoscopic repair for parastomal hernia using the sandwich technique. Indian J Surg 83(2):542–546

[23] Bertoglio C et al (2021) From keyhole to sandwich: change in laparoscopic repair of parastomal hernias at a single centre. Surg Endosc 35(4):1863–1871 32342214 10.1007/s00464-020-07589-2

[24] Dewulf M et al (2022) Robotic hernia surgery IV. English version: robotic parastomal hernia repair. Video report and preliminary results. Chirurgie (Heidelb) 93(Suppl 2):129140 10.1007/s00104-022-01779-5 PMC9747841 36480037

[25] Ayuso SA et al (2021) Robotic sugarbaker parastomal hernia repair: technique and outcomes. Hernia 25(3):809–815 33185770 10.1007/s10029-020-02328-x

